# Optimization of a bearing geometry for a cervical total disc replacement

**DOI:** 10.3389/fbioe.2025.1469366

**Published:** 2025-04-08

**Authors:** Lucia Kölle, Markus Flohr, Gregory Pryce, Andrew R. Beadling, Michael Bryant, Richard M. Hall, Stephen J. Ferguson, Benedikt Helgason

**Affiliations:** ^1^ Institute for Biomechanics, Department of Health Sciences and Technology, ETH Zürich, Zürich, Switzerland; ^2^ Medical Products Division, CeramTec GmbH, Plochingen, Germany; ^3^ Institute for Functional Surfaces, School of Mechanical Engineering, University of Leeds, Leeds, United Kingdom; ^4^ Department of Mechanical Engineering, School of Engineering, University of Birmingham, Birmingham, United Kingdom

**Keywords:** total disc replacement, ceramics, computational design, design optimization, spine

## Abstract

**Introduction:**

While Total Disc Replacements (TDRs) are generally performing well clinically, reoperation rates indicate that the full potential of the TDR concept might not have been reached. Inspired by the underlying complications related to biomechanics and material longevity that limit the performance of current TDRs, we propose a methodology for the development of TDR-bearings, that addresses such issues.

**Methods:**

Our methodology combines finite element model-based optimization with literature derived biomechanical data and an advanced ceramic material to design TDR-bearings. The design optimization aims to functionally replace the structures that are commonly excised (removed) or dissected (cut) during TDR implantation in the anterior column.

**Results:**

The optimized bearing geometry was able to replicate the moment-rotation curve of the anterior column of the natural C6/C7 level during coupled flexion/extension-anterior/posterior translation movement. Lateral bending and axial rotation were simulated to ensure the TDR would not fail during these load- and motion profiles. Experimental verification of the finite element model showed the suitability of our simulation approach.

**Discussion:**

The combination of computational techniques, advanced materials, and target biomechanical data may allow to overcome limitations of current TDRs and unlock the full potential of the TDR-concept.

## 1 Introduction

More than 300 million people world-wide suffered from neck pain for more than 3 months in 2015 (Hurwitz et al., 2018). Not only does this lower their quality of life, but it is also an economic burden due to loss of productivity. When conservative treatment of neck or arm pain fails for more than 6 weeks (Zechmeister et al., 2011), a surgical intervention may be indicated. One option is arthroplasty with a Total Disc Replacement (TDR). While TDRs generally perform well, considerable complication and reoperation rates have been reported. One meta-analysis study reported e.g., that within a 7-year follow-up, 5.2% of patients need reoperation at the index level, and 4.3% at adjacent levels (for fusion patients, it is 12.7%, respectively 10.8%) (Badhiwala et al., 2020).

The underlying complications can be divided into four non-exclusive classes: (1) TDR-biomechanics, especially a mismatch between asymptomatic and postoperative kinematics, such as motion loss (Zavras et al., 2022) or pain, part of which can be facet pain due to an abnormal shift of centre of rotation (COR) (Chen et al., 2017), or hypermobility (Blumenthal et al., 2024); (2), implant material and/or design deficiency, resulting in osteolysis due to wear (Parish et al., 2020; Zavras et al., 2022) or dislocation (Parish et al., 2020; Salari and McAfee, 2012), or metal allergy (Blumenthal et al., 2024); (3), surgical errors such as malpositioning (Zavras et al., 2022; Blumenthal et al., 2024) or improper sizing (Zavras et al., 2022); and (4) improper patient selection (Park et al., 2016; Skovrlj et al., 2015; Salari and McAfee, 2012).

Hypermobility is connected to TDR-biomechanics and may be a cause of accelerated facet degeneration and pain through its effect on facet loading (Kerferd et al., 2017). A speculated cause for hypermobility is resection of longitudinal ligaments (Kerferd et al., 2017). However, resection may be necessary to access the disc space, in case of the anterior longitudinal ligament, or to achieve sufficient decompression, in case of the posterior longitudinal ligament. In the spine, passive control of mobility might be represented in the nonlinearity of the anterior columns moment-rotation curves. Hypermobility may be connected to TDRs not replicating these nonlinear moment-rotation curves sufficiently. Therefore, it might be beneficial for TDRs to replicate the natural moment-rotation curves attempting to take over the function of the longitudinal ligaments and the IVD.

Furthermore, motion coupling is present in movements of the lower cervical spine. It appears intuitive that a TDR should enable the remaining anatomical structures (such as facet joints) to guide motions rather than compete with them. This could avoid damage of the anatomical structures, as well as the implant, and restore spinal biomechanics as closely as possible to the asymptomatic intact condition. While postoperative biomechanics have been commonly investigated *in vivo*, *ex vivo*, and *in silico* (Galbusera et al., 2008), and there are previous works on the optimization of TDR designs (Amadji et al., 2018; Zhou and Willing, 2019; 2020), the optimization of cervical TDRs to replicate the mechanical behaviour of the structures they replace, as well as addressing coupled motion, was not addressed in the literature so far.

The aim of the present study was to design a TDR-bearing geometry that replicates the moment-rotation curve of human C6/C7 anterior columns during coupled motions of flexion/extension-anterior/posterior translation. To this end, we optimized a bearing based on *ex vivo* moment-rotation curves of the anterior column (as intervertebral disc (IVD), anterior and posterior longitudinal ligaments are typically removed or damaged prior to TDR implantation) combined with *in vivo* instantaneous centre of rotation (ICR) data. The hypothesis was that the optimization of the TDR-bearing geometry will lead to a design that replicates the nonlinear moment-rotation curve of the structures that a TDR commonly replaces.

## 2 Materials and methods

### 2.1 Design optimization

#### 2.1.1 TDR design concept

**FIGURE 1:**
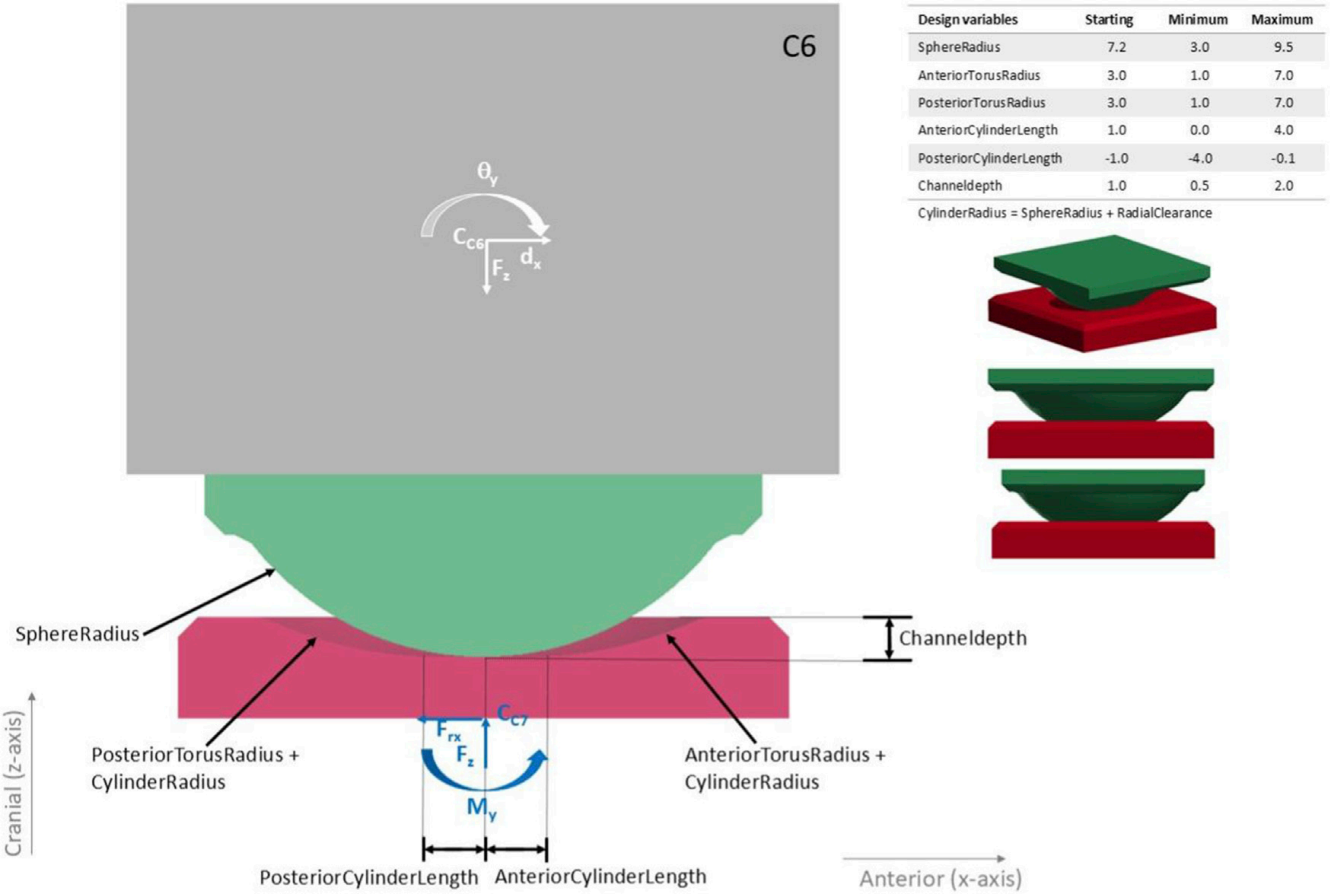
Sagittal cross-section through the finite element model of the baseline design with the geometric variables (baseline/initial values and their possible ranges) that were varied in the optimization. The superior TDR part is shown in green, the inferior TDR part in red and the C6 vertebra in grey, with the mesh size used during the optimization. The outputs that were read from the simulations were the moment in y-direction on the bottom of the TDR $M_{ry}$ and the superior vertebra’s rotation in y-direction $\theta_{y}.$ As in the experimental setup of (Hartman et al., 2016), the lower vertebra was fixed, the inferior-surface of the TDR was constrained in all six degrees of freedom in the FEM. Additional dependent variables were defined, for example, to adjust the anterior-posterior length of the superior endplate so that it does not limit the range of motion for smaller sphere radii but is large enough to offer a stable and manufacturable base for larger sphere radii. The smaller images on the right show further views of the baseline design.

Due to their advantageous properties, ceramics are used in TDRs (Kölle et al., 2022). The established bioceramic zirconia-toughened alumina material, BIOLOX®*delta* (CeramTec GmbH, Germany), was chosen, as it offers low wear (Döring et al., 2023), biocompatibility (Asif, 2018), high strength (Kuntz et al., 2009), MRI (Mödinger et al., 2023) and CT compatibility, and years of successful clinical use in hip arthroplasty (Alshammari et al., 2023). The design concept of the bearing geometry is based on toroidal and cylindrical shapes subtracted from the inferior part, and a spherical shape on the superior part of the TDR (Figure 1).

An implant height of 6 mm was chosen, as the mean disc height of C6/C7 was reported between 5.48 (Choi et al., 2017) and 6.4 mm (Yukawa et al., 2012). Appropriate implant height is necessary to enable a sufficient range of motion and avoid an increase of facet joint pressure (Wang et al., 2021). Dimensions of the vertebrae were taken from (Bazaldúa Cruz et al., 2011). A radial clearance of 0.07 mm was chosen. The baseline design, with which the optimization started, had a ball radius of 7.2 mm and torus radii of 3 mm inspired by the Prestige™ LP (Medtronic, United States) (Jung et al., 2013).

#### 2.1.2 Finite element model

A finite element model (FEM) was built in LS-PrePost V4.6.4 (LSTC, United States) containing only the necessary structures (Figure 1) to minimize computational cost. The effects of other structures are represented by applying the kinematics of the intact spine and by using the compressive forces predicted for the intact spine. The material parameters of BIOLOX®*delta* were provided by the manufacturer (CeramTec GmbH, Germany) and a linear elastic material model was used for the TDR with a density of 4.37 g/cm^3^, a Young’s modulus of 360 GPa and a Poisson`s ratio of 0.24. The friction coefficients for BIOLOX®*delta* on BIOLOX®*delta* in a lubricated condition were chosen as µ = 0.09 based on (Bishop et al., 2013; Haeussler and Pandorf, 2020).

A mesh sensitivity study showed negligible differences (percentage difference for the average absolute deviation of the moment: 0.75% in flexion and 0.88% in extension) between a mesh size of 0.25 and 0.5 mm (between 0.5 mm and 0.125 mm, it is 0.73% in flexion and 0.87% in extension). To reduce simulation time, the optimization was run with the larger mesh size (0.5 mm). As there is typically a hysteresis in the IVD’s moment-rotation curves, that the *ex vivo* data used in the objective does not contain (as they report only one way, likely neutral to deflection and not back to neutral), the moment was not recorded at the bottom of the TDR but 0.25 mm cranially, so that the TDRs hysteresis would be around the curve from literature. Following the optimization, a simulation of a full flexion/extension-anterior/posterior translation movement cycle, was run with smaller mesh size (0.25 mm) as it led to a smoother moment-rotation curve, and the moment at the bottom of the TDR was reported.

#### 2.1.3 Load, motion inputs and design objective

The moment-rotation curves of intact anterior columns were quantified based on the literature (Hartman et al., 2016), in which the kinematics of natural intact specimens were recorded before dissection and replayed. While the study reported rotation angles in relation to an ICR, it did not report the ICR itself. Therefore, the ICR-trajectory from a separate *in vivo* study (Anderst et al., 2013) was used in the present work. We support combining these datasets by the observation of Jonas et al. (Jonas et al., 2018), who found that ICRs of an *in vivo* study were comparable to those of an *ex vivo* cervical study, in which pure moments were applied to mono-segmental specimens. An *in silico study* (Bayoglu et al., 2019) reported axial compressive loads during movements based on musculoskeletal simulations as a percentage of bodyweight. In the present work, loads applied were scaled to the average bodyweight of an european adult: 70.8 kG (Walpole et al., 2012).

#### 2.1.4 Design optimization of the bearing geometry

The design objective was to minimize the mean square error (MSE) between the Y-moment-rotation curve of the TDR (Y-axis pointing laterally) and that of C6/C7 anterior columns replaying the kinematics of the intact functional spinal units (FSUs). The ICR-trajectory was used to calculate the displacements (rotational and translational) that were applied to the vertebra superior of the TDR to which also the scaled load curve was applied.

The corresponding optimization problem can be written as (Equations 1a, 1b, 1c):

$$\min_{p}\text{ MSE}\left(p\right)$$ (1a)

$$s.t.\;\;\;p\in \Omega \;\left(\text{design space}\right)$$ (1b)

$$\sigma \left(p\right)\leq \sigma_{\max}\;\left(\text{stress constraint}\right)$$ (1c)

where p is a vector corresponding to the geometrical optimization variables: *SphereRadius*, *AnteriorCylinderLength*, *PosteriorCylinderLength*, *AnteriorTorusRadius*, *PosteriorTorusRadius*, *Channeldepth* (see Figure 1). The design space Ω imposes direct bounds on the geometric variables transcribing constraints regarding manufacturing and anatomy. Additionally, multiple dependent variables and sampling constraints were defined. Finally, σ(p) ≤ σ_max_ imposes bounds on the maximal principal stresses during the simulations (σ_max_ = 0.3 GPa, based on the biaxial flexural strength of “extra-high strength” zirconia-toughened alumina ceramics from ISO 6472 Part 2 (2019) and a safety factor of 2) to prevent material failure. The implicit finite element simulations consisted of transient stress initializations followed by simulations of flexion and extension motions coupled with anterior/posterior translation.

**FIGURE 2:**
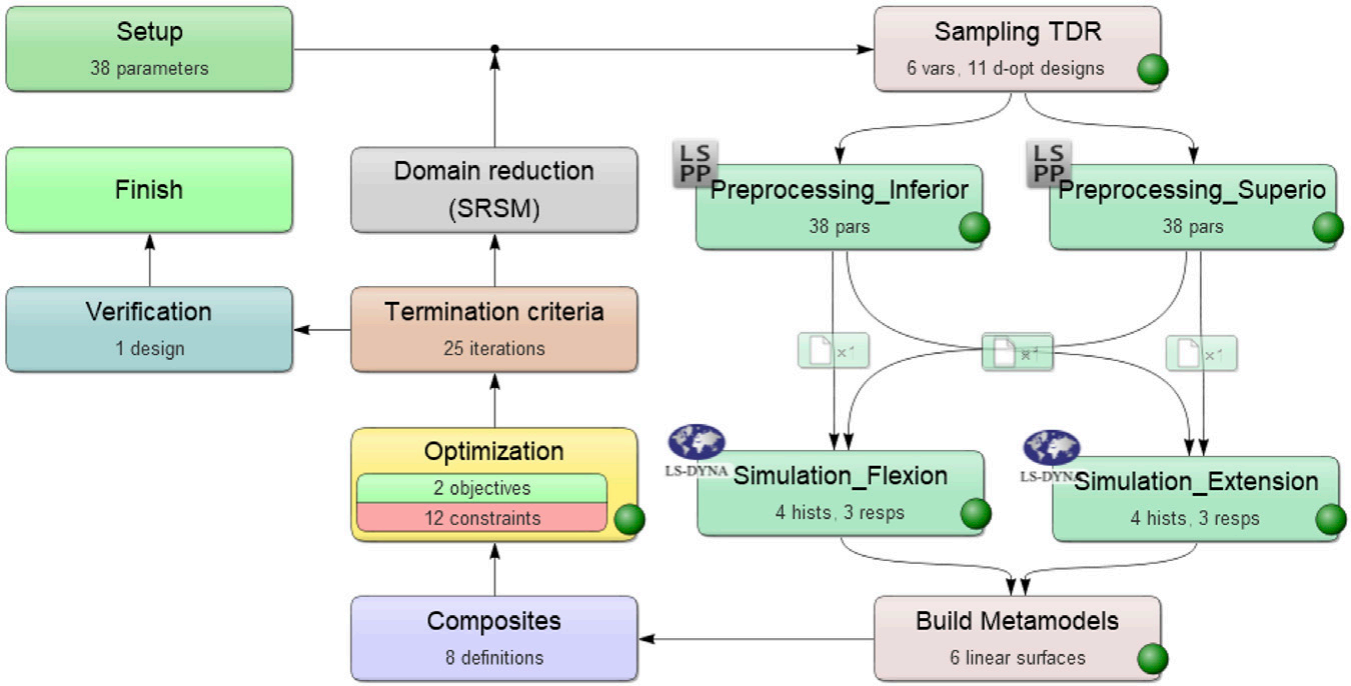
Flow chart for the optimization process in LS-OPT (LSTC, United States).

Figure 2 shows the optimization setup in LS-OPT V7.0.0 (LSTC, United States). A baseline design and variations of it were created (sampling- and pre-processing stages) and their performance during the simulations (simulation stage) was used to create metamodels (metamodel stage). Based on this, the parameter ranges were narrowed (domain-reduction stage) so that in the next iteration, sampling was done only in the expedient parts of the initial ranges. This way, the design parameters were narrowed down until one of the termination criteria was reached. This optimization strategy is also known as sequential metamodel based optimization with domain reduction. Polynomial metamodels of linear order and D-optimal point selection (maximization of determinant of moment matrix of least squares formulation) of eleven design versions per iteration were selected, as well as the Leapfrog optimizer for constrained minimization algorithm. Termination criteria were set to a design change tolerance of 1e-3, an objective function tolerance of 1e-4 and a maximum of 25 iterations. Reaching any of these criteria was sufficient for termination. The metamodels are generated based on the MSE computed in LS-OPT.

LS-Dyna version R13.0, LS-OPT V7.0.0 and LS-PrePost V4.6.4 (all software from LSTC, United States) were used for the optimization that was run on a computer cluster with 60 cores and 320 GB RAM.

#### 2.1.5 Computational investigation of the optimized design

A sensitivity analysis evaluated robustness of the optimized design to uncertainties in the input data. Therefore, the superior-inferior and anterior-posterior trajectories of the ICR were varied within a 95% confidence interval, the friction coefficient and patient-bodyweight were varied between 50% and 150% of the aforementioned values. The maximal MSEs were calculated.

Furthermore, maximal axial rotation and lateral bending based on Hartman et al. (2016) were combined with compressive forces during these movements reported in Bayoglu et al. (2019) and simulated to evaluate whether the stress limits of the ceramic were violated.

### 2.2 Experimental verification of the simulations

**FIGURE 3:**
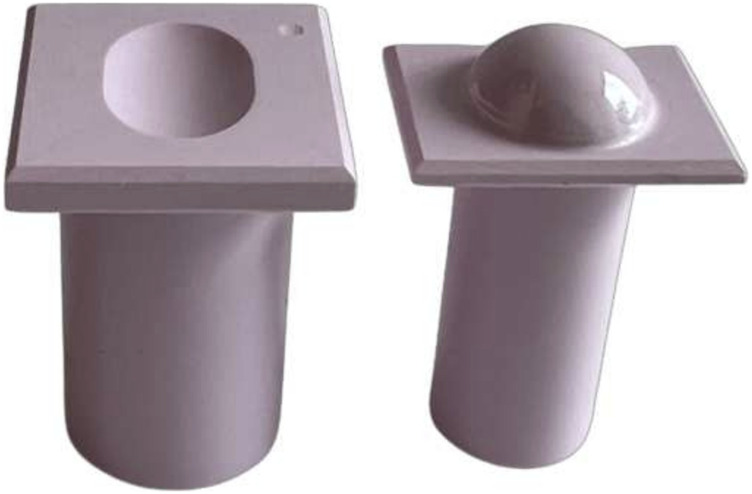
Samples of the optimized bearing geometries - view from anterior. Left: inferior part. The deepening in one corner is a mark to identify the posterior side of the sample. Right: superior part. The cylinders provide secure mounting of the samples in the testing device. Sample manufacturing was done as follows: Forming from raw material, green machining (milling), sintering, hot isostatic pressing, sintering, polishing.

Six samples with the optimized bearing geometries were manufactured from BIOLOX®*delta*, and their bearing surfaces were polished (Figure 3). Samples were cleaned in de-ionized water in an ultrasonic bath and then dried with an airgun. After they were mounted in the simulators fixtures, their bearing surfaces were cleaned again using acetone and lab wipes.

**FIGURE 4:**
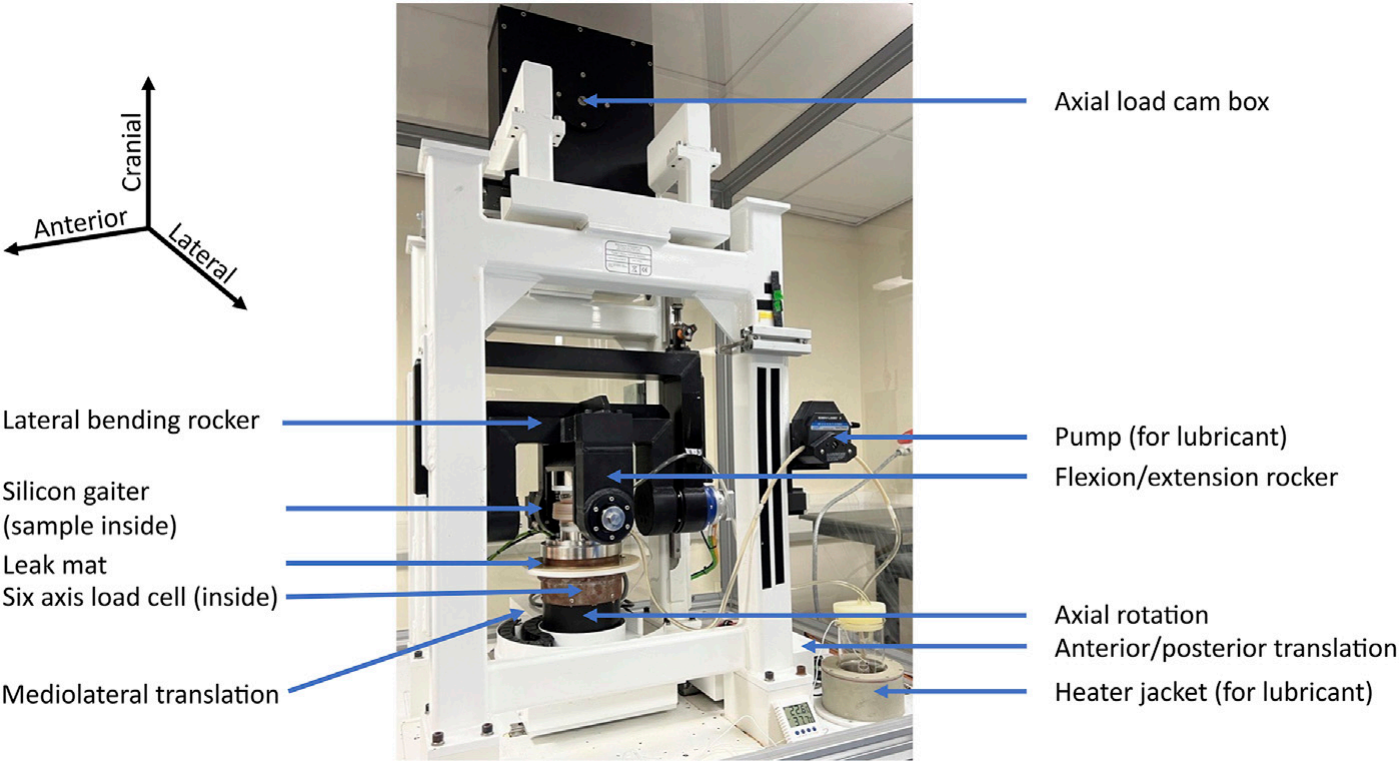
Universal joint simulator. The sample is mounted in fixtures within the silicon gaiter, where it is submerged in lubricant (diluted calf serum).

Testing was conducted using a universal joint simulator (Prosim 1-Station Universal Simulator, Simulation Solutions Ltd., United Kingdom) (Figure 4). Equivalent load and motion inputs were used as in the simulation. However, the overall situation was not completely equivalent, as described at the beginning of the next paragraph. Six samples were tested submerged in diluted calf serum [20 g ± 2 g protein/l, (GE Healthcare Lifesciences, United States), 0.03% sodium azide (Severn Biotech Ltd., United Kingdom) diluted with de-ionized water] at 37°C (±2°C) based on ISO 18192-1:2011. For each sample, 200 cycles were run, and the duration of one cycle was 3000 ms, which is equivalent to the movement speed of the FEM simulations and based on an *in vivo* study (Anderst et al., 2013). Cycles 180-189 (mean) were evaluated to avoid run-in and run-out phenomena as well as to use more representative data than using a single cycle.

#### 2.2.1 Simulation of the verification experiment

**FIGURE 5:**
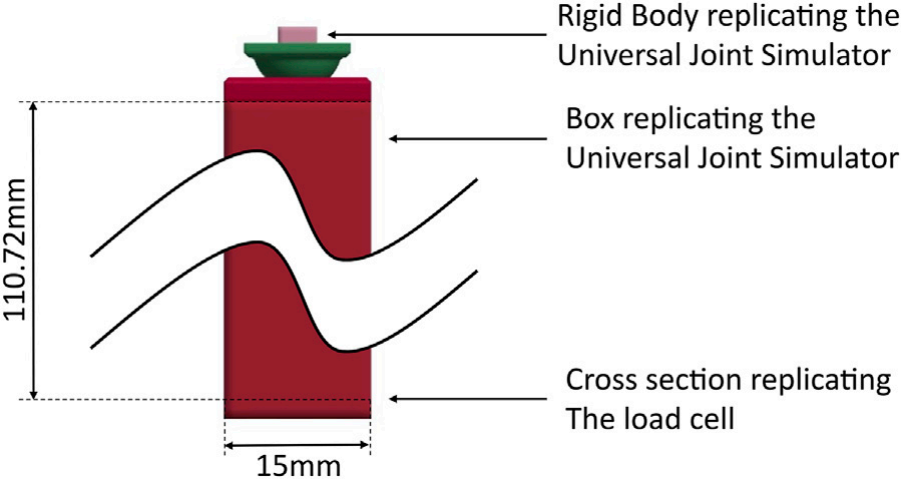
Simulation of the experiment, superior part 0.45 mm more posterior than in the optimization simulations. The simulation model contains the TDR sample and a rigid body with the centre defined in the centre of rotation of the universal joint simulator to apply rotations to the sample consistent with the experiment.

It was not possible to apply the vertical load in the universal joint simulator in the same way it was applied in the optimization (application to the vertebra above the implant (optimization) instead of to the centre of the spherical part (experiment)). This affects the moment, as the load application point is deflected more during coupled rotations, leading to a longer lever arm. The load- and motion profiles and application point from the experiment were applied to a FEM (virtual test bed) including the average geometry of the specimens tested, and read out at the position of the loadcell; to compare the measured and simulated responses (Figure 5). The sample geometries were based on geometry measurements using an optical measuring device GOM (ATOS III, Carl Zeiss GOM Metrology GmbH, Germany) prior to any testing.

Load and displacement outputs from the universal joint simulator (mean of cycles 180–189) were used as simulation inputs. This accounts for imperfections of the simulators controls in meeting the desired profiles and smoothing of the profiles for the simulator input. Mesh size (0.0625 mm), initial and maximal timestep (both 1 ms instead of 10 ms) were reduced compared to previous simulations.

Experimental testing of pure flexion/extension under static load was performed on five samples to measure the dynamic friction coefficient of the samples in the tested condition. This friction coefficient was used in the simulation of the experiment.

## 3 Results

### 3.1 Design optimization

#### 3.1.1 Design optimization of the bearing geometry

**FIGURE 6:**
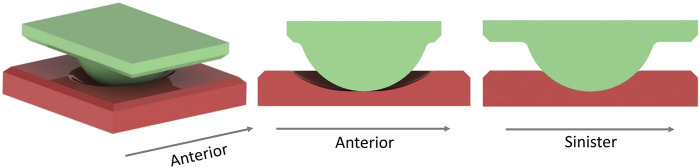
Posterolateral view (left), sagittal cross section (centre) and coronal cross section (right) of the optimized design.

**FIGURE 7:**
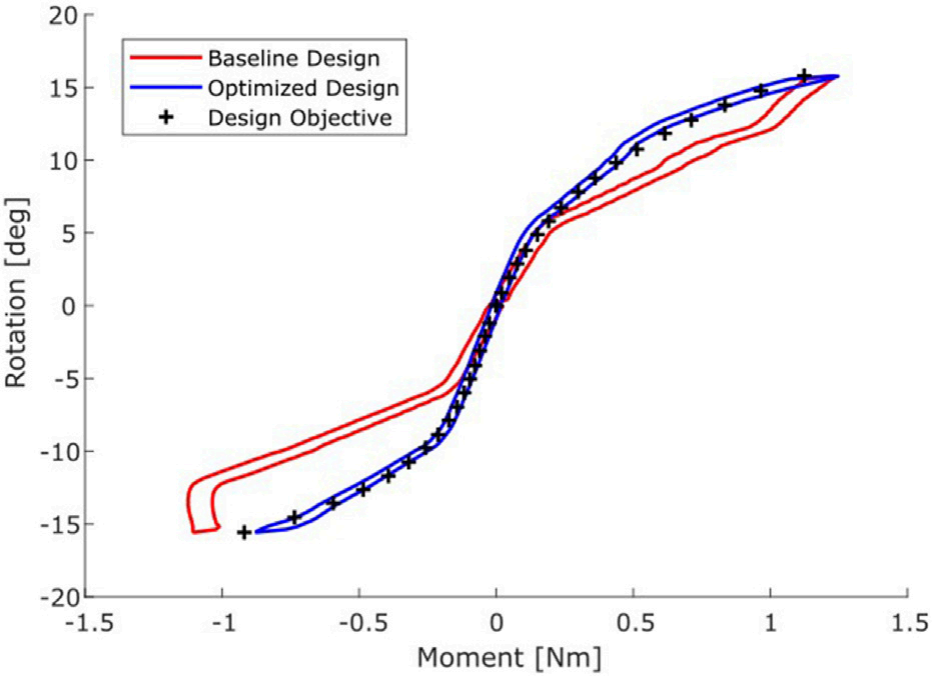
Moment-Rotation curves of the baseline (red) and optimized design (blue) compared to the target moment-rotation response (black +).

The optimization reached a termination criterion after 24 iterations, within 5.5 h. The optimized design parameters are given in Table 1, the design is shown in Figure 6. There are clear differences between the parameters of the baseline and optimized design. When performing a coupled flexion/extension-anterior/posterior translation movement, the moment-rotation curve exhibits nonlinearity and hysteresis (Figure 7) as does the moment-rotation curve of an IVD (Newell et al., 2017). Especially in extension, the behaviour of the optimized design matches that of the design objective considerably better than the baseline design (Figure 7). The design optimization achieved a 99% reduction of the design objective (MSE) compared to the baseline design.

**TABLE 1:** Design variables of the baseline and optimized design and the design space. See Figure 1 for variable definitions. All values in mm.

Design variables	Starting	Minimum	Maximum	Optimized
*SphereRadius*	7.2	3.0	9.5	4.605
*AnteriorTorusRadius*	3.0	1.0	7.0	2.309
PosteriorTorusRadius	3.0	1.0	7.0	1.823
*AnteriorCylinderLength*	1.0	0.0	4.0	0.681
*PosteriorCylinderLength*	−1.0	−4.0	−0.1	−1.157
Channeldepth	1.0	0.5	2.0	1.459

#### 3.1.2 Computational investigation of the optimized design

In axial rotation and lateral bending, the maximal principal stresses were 0.05 GPa (min: −0.12) and 0.06 GPa (min: −0.10), which is within the bounds of the stress constraints of −0.3 ≤ σ(p) ≤ 0.3 GPa. The sensitivity analysis showed that bodyweight and superior-inferior ICR-trajectory variation have the strongest effect on moment-rotation curves with maximal MSEs of 0.04 and 0.04 ${Nm}^{2}$ . For anterior-posterior ICR-trajectory and friction coefficient variation, the maximal MSEs were 0.002 and 1.55e-04 ${Nm}^{2}.$ However, a direct comparison of these values is not possible, as for the ICR-parameters, variation was investigated between ±2 SD, and for the other between 50% and 100%. The study from which the bodyweight was taken did not provide SD.

### 3.2 Experimental verification of the simulations

**FIGURE 8:**
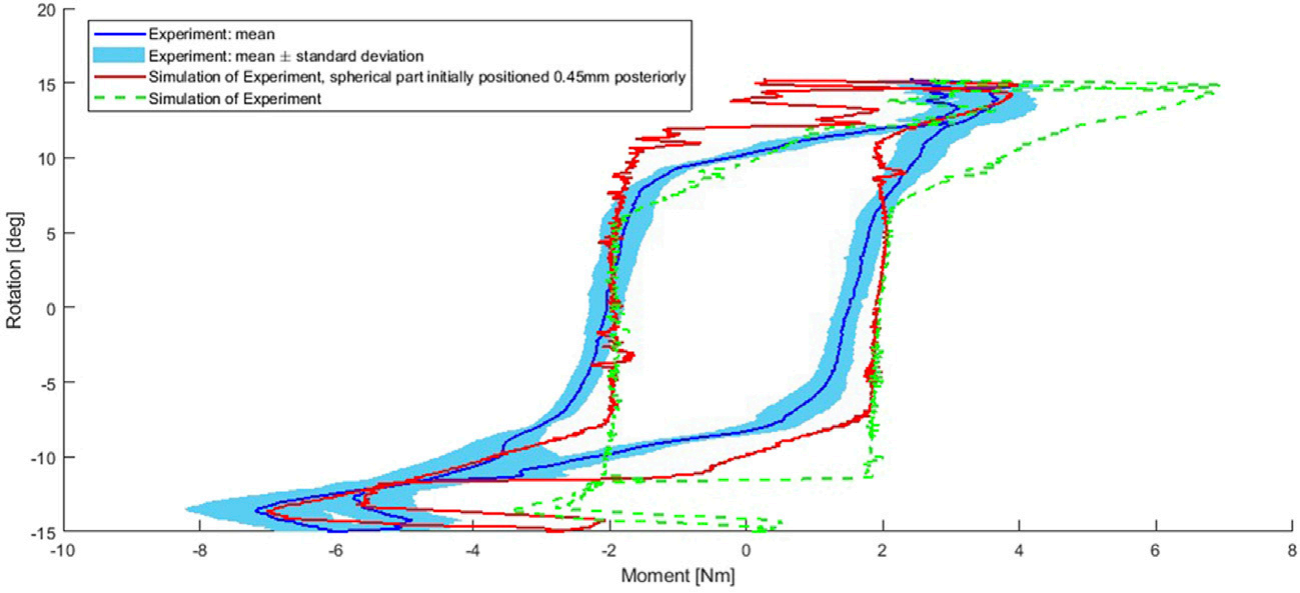
Experiment and simulation of the experiment. Simulation: both for the intended initial placement of the superior implant part and the superior implant part mounted 0.45 mm posteriorly to the intended position. Simulation outputs filtered using a moving average filter of 4 points.

The magnitude of the moment in the experiment was larger than in the optimization, this was partially due to there being a considerably longer lever arm to the loadcell than to the location of the read out in the optimization. The absolute moment was larger in extension than in flexion, this was likely to be connected to the anterior-posterior relative placement of the TDR parts to each other or may be connected to sliding. Figure 8 shows the experimental result, including the mean and the envelope containing one standard deviation.

#### 3.2.1 Simulation of the verification experiment

The simulation of the experiment produced moment-rotation curves that are comparable to the experiment (Figure 8). The moment-rotation curves are sensitive to the initial relative positioning of the two implant parts to each other in anterior-posterior direction (Figure 8 - red). Initial placement of the superior implant part 0.45 mm more posterior leads to a better match with the experimental curve and is in line with video data of an initial dry experiment with a test sample when overlaying the video and an animation video of the simulation. The peak moment of the experiment differed by 0.036 Nm (0.92%) in flexion and 0.17 Nm (2.38%) in extension from the simulation of the experiment when taking the 0.45 mm into account (simulation data filtered using moving average filter of 4). However, after the maximal flexion and extension, the simulation had a strong change in the moment without changing the rotation as much as in the experiment. Furthermore, the central parts of the simulation curve are more linear than in the experiment.

Geometric measurements of the samples prior to any testing showed that the main difference between the optimized design and as-manufactured samples was a larger radial clearance of the samples.

## 4 Discussion

The aim of the present study was to design a bearing geometry for a TDR that replicates the moment-rotation curve of human C6/C7 anterior columns during coupled flexion/extension-anterior/posterior translation motion. The bearing was successfully optimized using an *ex vivo* measured moment-rotation curve of anterior columns and *in vivo* measured ICR data. This resulted in a design that confirmed our hypothesis, that it is possible through geometric optimization of an articulating TDR bearing design to replicate the nonlinear moment-rotation curve of the natural intact anterior column.

The MSE of the optimized design was reduced by 99% compared to the baseline design. The baseline design was symmetric in the anterior-posterior direction. However, segmental kinematics are not, nor is the optimized design. While previous literature has reported computational optimization of TDRs (Amadji et al., 2018; Zhou and Willing, 2019; Zhou and Willing, 2020), there was no other work in literature that optimized the same design-type or used the same design objective for a TDR, nor used coupled motions. Other studies have reported non-linear moment rotation curves with hysteresis for articulating (Patwardhan and Havey, 2020) and non-articulating TDRs (Patwardhan and Havey, 2020; Guyer et al., 2018) in cadaveric specimens.

Simulation of lateral bending and axial rotation movements predicted no fracture risk of the bearing, also during these motions. Sensitivity analysis implied that the design’s biomechanical signature is quite insensitive to variations in ICR trajectory, loading/bodyweight and friction coefficient. It is notable that superior-inferior ICR-trajectory variation was one of the factors with the strongest effect, since a significant cranial shift of the COR was reported for a ball-in-trough TDR (Hu et al., 2019). While this study did not report pain issues, another suggested that an abnormal shift of the COR has been connected to increased facet pain (Chen et al., 2017). As for the ICR, a cranial shift of the COR is caused by reduced anterior translation (Hu et al., 2019).

Experiments were performed to verify the simulations. While the experiment had to be compared to a simulation of the experiment rather than to the simulations of the optimization directly, this general verification was successful. Comparison of experimental and FEM-derived results indicates that the simulations are reliable. The peak moment in the simulation of the experiment differed by less than 2.5% from the experiment (when assuming 0.45 mm anterior-posterior misplacement of the implant parts relative to each other). We therefore conclude that the model error most likely does not have clinical relevance. One possible cause of discrepancy is the use of an approximate FEM rather than modelling the exact geometries of the individual samples. For example, that the central part of the simulation response is more linear might be because the FEM assumes the central part of the inferior bearing part to be the negative of a cylinder when in reality there is some imperfection. In a series production, manufacturing imperfections would be a fraction of what they were in this study, therefore this would not be an issue. The friction-model in the FEMs does not depend on the slide-roll ratio, which may affect the moment-rotation curves behaviour following maximal flexion and extension. The universal joint simulator that was used was not calibrated for displacements which might have caused the closer match to the experimental data when positioning the TDR parts in 0.45 mm anterior-posterior distance to each other. The universal joint simulator was furthermore not optimized for the small loads and displacements in the cervical spine and the uncertainties of the loadcell and simulator are considerable (±50 N for axial force; ±0.15 Nm for torque and ± 0.008 deg for rotation in flexion/extension). The simulation of the experiment was sensitive to mesh size and timestep and at a larger computational cost, a closer match between the experiment and the simulation of the experiment may have been achieved, or if the individual samples geometries would have been modeled instead of a mean geometry of the tested samples.

Limitations of the optimization were the combination of different studies for the optimization-input, especially loading that is not identical to the load in the study reporting *in vitro* moment-rotation curves. Also, the optimization used a friction coefficient that was lower than the one found in the experiment. This may be connected to the literature value being based on hip replacements, which might not be representative of the small bearings of TDRs, especially since it was a different design-type, and radial clearance of the samples being larger than planned initially.

The innovative contribution of this work is the methodology that allows to leverage existing biomechanical data, an advanced ceramic biomaterial and computational design optimization to address function-related complications currently limiting the full potential of the TDR concept. Recent technological advances allow us to develop a comprehensive and systematic approach, directly comparing many designs in digital tests and using selected physical *in vitro* tests to verify the simulations. These physical tests are conducted under established conditions built on decades of expertise developed in implant-testing. Connecting these advancements from diverse research fields seems promising, and such methodologies can be transferred to other applications such as knee or shoulder arthroplasty. The efficient optimization process described could be used to explore the potential of design concept options in product development, or in the development of patient-specific implants.

## 5 Conclusion

We present a methodology for the optimization of bearing geometries that produce a nonlinear moment-rotation response similar to that of an intact natural anterior column. A close match between the design objective and the optimization results in the simulations was achieved. Geometric optimization resulted in a bearing that is asymmetric in anterior-posterior direction. To verify these simulations, samples were produced, and experiments were conducted *in vitro,* demonstrating a good correspondence between simulation and experiment results. We conclude that computational design optimization can be used to efficiently generate a bearing geometry for a TDR that replicates the moment-rotation curve of an anterior column during coupled motion of flexion/extension-anterior/posterior translation. Constraints ensure manufacturability and durability. Simulations of lateral bending and axial rotation indicated reliability. The clinical relevance and contribution of this publication mainly lies in the methodology, which can be adjusted for different design objectives and different applications. Computational design and optimization have potential in application for complex systems such as orthopaedic implants. With this, the design of new devices or optimization of existing devices can be performed efficiently, specifically addressing concerns stemming from clinical experience and building on design concepts inspired by spinal biomechanics.

## Data Availability

The original contributions presented in the study are included in the article/Supplementary Material, further inquiries can be directed to the corresponding author.

## References

[B1] Alshammari M. O. de Petrillo G. Epure L. M. Huk O. L. Zukor D. J. Antoniou J. (2023). Outcomes of ceramic-on-ceramic bearing total hip arthroplasty: a minimum 10-year follow-up study. J. Arthroplasty 38, S146–S151. 10.1016/j.arth.2023.04.018 37084924

[B2] Amadji M. Ameddah H. Mazouz H. (2018). Numerical shape optimization of cervical spine disc prosthesis prodisc-C. J. Biomimetics, Biomater. Biomed. Eng. 36, 56–69. 10.4028/www.scientific.net/JBBBE.36.56

[B3] Anderst W. Baillargeon E. Donaldson W. Lee J. Kang J. (2013). Motion path of the instant center of rotation in the cervical spine during *in vivo* dynamic flexion-extension. Spine (Phila. Pa. 1976) 38, E594–E601. 10.1097/BRS.0b013e31828ca5c7 23429677 PMC3656913

[B4] Asif I. M. (2018). Characterisation and biological impact of wear particles from composite ceramic hip replacements. Univ. Leeds - Sch. Mech. Eng. 309. Available online at: http://etheses.whiterose.ac.uk/20563/.

[B5] Badhiwala J. H. Platt A. Witiw C. D. Traynelis V. C. (2020). Cervical disc arthroplasty versus anterior cervical discectomy and fusion: a meta-analysis of rates of adjacent-level surgery to 7-year follow-up. J. Spine Surg. 6, 217–232. 10.21037/jss.2019.12.09 32309660 PMC7154351

[B6] Bayoglu R. Galibarov P. E. Verdonschot N. Koopman B. Homminga J. (2019). Twente Spine Model: a thorough investigation of the spinal loads in a complete and coherent musculoskeletal model of the human spine. Med. Eng. Phys. 68, 35–45. 10.1016/j.medengphy.2019.03.015 31010615

[B7] Bazaldúa Cruz J. J. González Larios A. Gómez Sánchez A. Villarreal Silva E. E. Velázquez Gauna S. E. Sánchez Uresti A. (2011). Morphometric study of cervical vertebrae C3-C7 in a population from northeastern Mexico. Int. J. Morphol. 29, 325–330. 10.4067/S0717-95022011000200003

[B8] Bishop N. E. Hothan A. Morlock M. M. (2013). High friction moments in large hard-on-hard hip replacement bearings in conditions of poor lubrication. J. Orthop. Res. 31, 807–813. 10.1002/jor.22255 23239536

[B9] Blumenthal S. L. Ohnmeiss D. D. Courtois E. C. Guyer R. D. Zigler J. E. Shellock J. L. (2024). Treatment of failed cervical total disc replacements in a series of 53 cases and description of a management strategy. Eur. Spine J. 33, 3117–3123. 10.1007/s00586-024-08402-7 39026079

[B10] Chen C. Zhang X. Ma X. (2017). Durability of cervical disc arthroplasties and its influence factors. Med. Baltim. 96, e5947. 10.1097/MD.0000000000005947 PMC5312992 28178135

[B11] Choi S. H. Lee H. Cho J. H. Jung J. I. Lee D.-H. (2017). Radiological parameters of undegenerated cervical vertebral segments in a Korean population. Clin. Orthop. Surg. 9, 63. 10.4055/cios.2017.9.1.63 28261429 PMC5334029

[B12] Döring J. Buchholz A. Herbster M. Gehring J. Betke U. Chodór P. (2023). Damage analysis of retrieved Biolox®delta components used in hard and soft bearings. Acta Biomater. 158, 827–842. 10.1016/j.actbio.2022.12.055 36599400

[B13] Galbusera F. Bellini C. M. Brayda-Bruno M. Fornari M. (2008). Biomechanical studies on cervical total disc arthroplasty: a literature review. Clin. Biomech. 23, 1095–1104. 10.1016/j.clinbiomech.2008.06.002 18635294

[B14] Guyer R. D. Voronov L. I. Havey R. M. Khayatzadeh S. Carandang G. Blank K. R. (2018). Kinematic assessment of an elastic-core cervical disc prosthesis in one and two-level constructs. Jor Spine 1, e1040. 10.1002/jsp2.1040 31463455 PMC6686807

[B15] Haeussler K. Pandorf T. (2020). Lever-out moments of a lipped ceramic liner. Orthop. Proc. 102-B, 90. 10.1302/1358-992X.2020.2.090

[B16] Hartman R. A. Tisherman R. E. Wang C. Bell K. M. Lee J. Y. Sowa G. A. (2016). Mechanical role of the posterior column components in the cervical spine. Eur. Spine J. 25, 2129–2138. 10.1007/s00586-016-4541-1 27052405

[B17] Hu X. Jiang M. Liu H. Rong X. Hong Y. Ding C. (2019). Five-year trends in center of rotation after single-level cervical arthroplasty with the prestige-LP disc. World Neurosurg. 132, e941–e948. 10.1016/j.wneu.2019.07.042 31302269

[B18] Hurwitz E. L. Randhawa K. Yu H. Côté P. Haldeman S. (2018). The Global Spine Care Initiative: a summary of the global burden of low back and neck pain studies. Eur. Spine J. 27, 796–801. 10.1007/s00586-017-5432-9 29480409

[B19] Jonas R. Demmelmaier R. Hacker S. P. Wilke H.-J. (2018). Comparison of three-dimensional helical axes of the cervical spine between *in vitro* and *in vivo* testing. Spine J. 18, 515–524. 10.1016/j.spinee.2017.10.065 29074465

[B20] Jung T.-G. Woo S.-H. Park K.-M. Jang J.-W. Han D.-W. Lee S. J. (2013). Biomechanical behavior of two different cervical total disc replacement designs in relation of concavity of articular surfaces: ProDisc-C® vs. Prestige-LP®. Int. J. Precis. Eng. Manuf. 14, 819–824. 10.1007/s12541-013-0107-x

[B21] Kerferd J. W. Abi-Hanna D. Phan K. Rao P. Mobbs R. J. (2017). Focal hypermobility observed in cervical arthroplasty with Mobi-C. J. Spine Surg. 3, 693–696. 10.21037/jss.2017.08.19 29354749 PMC5760431

[B22] Kölle L. Ignasiak D. Ferguson S. J. Helgason B. (2022). Ceramics in total disc replacements: a scoping review. Clin. Biomech. 100, 105796. 10.1016/j.clinbiomech.2022.105796 36435073

[B23] Kuntz M. Masson B. Pandorf T. (2009). “Current state of the art of the ceramic composite material BIOLOX®DELTA,” in Strength of materials (Hauppauge, NY: Nova Science Publishers), 133–155.

[B24] Mödinger Y. Anttila E. D. Baker G. M. Gross D. C. Porporati A. A. (2023). Magnetic resonance safety evaluation of a novel alumina matrix composite ceramic knee and image artifact comparison to a metal knee implant of analogous design. Arthroplast. Today 22, 101170. 10.1016/j.artd.2023.101170 37521740 PMC10374871

[B25] Newell N. Little J. Christou A. Adams M. Adam C. Masouros S. (2017). Biomechanics of the human intervertebral disc: a review of testing techniques and results. J. Mech. Behav. Biomed. Mater. 69, 420–434. 10.1016/j.jmbbm.2017.01.037 28262607

[B26] Parish J. M. Asher A. M. Coric D. (2020). Complications and complication avoidance with cervical total disc replacement. Int. J. Spine Surg. 14, S50–S56. 10.14444/7091 32994306 PMC7528766

[B27] Park J.-B. Chang H. Yeom J. S. Suk K.-S. Lee D.-H. Lee J. C. (2016). Revision surgeries following artificial disc replacement of cervical spine. Acta Orthop. Traumatol. Turc. 50, 610–618. 10.1016/j.aott.2016.04.004 27939974 PMC6197355

[B28] Patwardhan A. G. Havey R. M. (2020). Prosthesis design influences segmental contribution to total cervical motion after cervical disc arthroplasty. Eur. Spine J. 29, 2713–2721. 10.1007/s00586-019-06064-4 31309331

[B29] Salari B. McAfee P. C. (2012). Cervical total disk replacement: complications and avoidance. Orthop. Clin. North Am. 43, 97–107. 10.1016/j.ocl.2011.08.006 22082633

[B30] Skovrlj B. Lee D.-H. Caridi J. M. Cho S. K.-W. (2015). Reoperations following cervical disc replacement. Asian Spine J. 9, 471–482. 10.4184/asj.2015.9.3.471 26097667 PMC4472600

[B31] Walpole S. C. Prieto-Merino D. Edwards P. Cleland J. Stevens G. Roberts I. (2012). The weight of nations: an estimation of adult human biomass. BMC Public Health 12, 439. 10.1186/1471-2458-12-439 22709383 PMC3408371

[B32] Wang X.-F. Meng Y. Liu H. Wang B.-Y. Hong Y. (2021). The impact of different artificial disc heights during total cervical disc replacement: an *in vitro* biomechanical study. J. Orthop. Surg. Res. 16, 12. 10.1186/s13018-020-02157-9 33407705 PMC7789724

[B33] Yukawa Y. Kato F. Suda K. Yamagata M. Ueta T. (2012). Age-related changes in osseous anatomy, alignment, and range of motion of the cervical spine. Part I: radiographic data from over 1,200 asymptomatic subjects. Eur. Spine J. 21, 1492–1498. 10.1007/s00586-012-2167-5 22310883 PMC3535253

[B34] Zavras A. G. Sullivan T. B. Singh K. Phillips F. M. Colman M. W. (2022). Failure in cervical total disc arthroplasty: single institution experience, systematic review of the literature, and proposal of the RUSH TDA failure classification system. Spine J. 22, 353–369. 10.1016/j.spinee.2021.08.006 34419625

[B35] Zechmeister I. Winkler R. Mad P. (2011). Artificial total disc replacement versus fusion for the cervical spine: a systematic review. Eur. Spine J. 20, 177–184. 10.1007/s00586-010-1583-7 20936484 PMC3030712

[B36] Zhou C. Willing R. (2019). Development of a biconcave mobile-bearing lumbar total disc arthroplasty concept using finite element analysis and design optimization. J. Orthop. Res. 37, 1805–1816. 10.1002/jor.24315 31042323

[B37] Zhou C. Willing R. (2020). Multiobjective design optimization of a biconcave mobile-bearing lumbar total artificial disk considering spinal kinematics, facet joint loading, and metal-on-polyethylene contact mechanics. J. Biomech. Eng. 142, 041006. 10.1115/1.4045048 31574140

